# Predictors of Staying at Home during the COVID-19 Pandemic and Social Lockdown based on Protection Motivation Theory: A Cross-Sectional Study in Japan

**DOI:** 10.3390/healthcare8040475

**Published:** 2020-11-11

**Authors:** Tsuyoshi Okuhara, Hiroko Okada, Takahiro Kiuchi

**Affiliations:** Department of Health Communication, School of Public Health, The University of Tokyo, Tokyo 113-8655, Japan; okadahiroko-tky@umin.ac.jp (H.O.); tak-kiuchi@umin.ac.jp (T.K.)

**Keywords:** COVID-19, novel coronavirus, social lockdown, protection motivation theory, health behavior, health communication

## Abstract

During the COVID-19 pandemic, a social lockdown should be put in place and individuals should stay at home. Behavioral change is the only way to prevent the pandemic and overwhelmed healthcare systems until vaccines are available. We aimed to examine the psychological factors that predict staying at home during the COVID-19 pandemic and social lockdown. A total of 1980 participants in Japan completed a survey for this study from 9 to 11 May 2020, when the state of emergency covered all prefectures in the country. Self-reported behavior in terms of staying at home, the perceived severity of the pandemic, vulnerability to the pandemic, response efficacy, and self-efficacy based on protection motivation theory were assessed. Multiple regression analysis showed that perceived severity (standardized β = 0.11, *p* < 0.001) and self-efficacy (standardized β = 0.32, *p* < 0.001) significantly predicted greater levels of staying at home, after controlling for socio-demographics. However, perceived vulnerability and response efficacy did not. To encourage people to stay at home during the pandemic and social lockdown, increasing the perceived severity of infection by COVID-19 and self-efficacy in terms of exercising restraint with respect to going out may consequently encourage people to stay at home.

## 1. Introduction

The outbreak of the coronavirus disease (COVID-19) has emerged as the largest global pandemic in recent history [1]. Currently, no medicine has been identified for the treatment of the disease [2]. COVID-19 is a transmissible disease that can be passed from an infected individual through any object that carries the virus to anyone who comes into contact with it. Therefore, social lockdown has been recommended by health experts as a preventive measure [3,4]. Social lockdown is a restriction of inter-individual physical contacts [5]. Under social lockdown, unnecessary movements and contacts between individuals are not allowed. Experts have proposed that social lockdown would generate better consequences, such as controlling the increase in the number of infected individuals, restricting community infection, preventing the healthcare system from becoming overloaded, and providing better health care to the infected individuals [5]. Owing to its importance, the governments of many countries across the world have declared local and national social lockdowns [5,6].

In Japan, the government declared a state of emergency on 7 April 2020 [7]. The state of emergency allowed the prefectural governors to request residents to refrain from unnecessary and non-urgent outings, and also to request business operators to restrict their use of stores and facilities [7]. Initially, the state of emergency covered the period from 8 April 2020 to 6 May 2020, and the target area was seven prefectures, including Tokyo and Osaka [7]. As of 7 April 2020, the number of infected individuals was 4625, and the number of deaths was 80 [8]. Those numbers rapidly increased after the declaration, rising to 14,034 cases and 170 deaths as of 15 April 2020 [8]. Therefore, on 16 April 2020, the area covered by the state of emergency was expanded to all prefectures in Japan [9]. Moreover, the Japanese government designated “specified warning prefectures” where there had been a particular increase in the number of infected individuals. There were six “specified warning prefectures”, including Hokkaido and Kyoto, in addition to the seven prefectures targeted first [9]. Furthermore, on 2 May 2020, the target period was extended to 31 May 2020 [9].

However, the Japanese state of emergency had few legally enforceable measures [10], and it was not a legal lockdown of the type imposed in some countries [11]. The Japanese stay-at-home order was just a “request” by the governors, and they had no legal powers to penalize individuals who disregarded calls to stay at home [11]. Accordingly, only about 40% of individuals reduced their frequency of shopping trips, and only about 30% reduced outing for the purpose of commuting in the seven prefectures first targeted in the state of emergency between a week and ten days after the declaration of emergency [12]. Even in countries in Europe and states in the U.S. where the lockdown had legal force, some people resisted it and disregarded calls to stay at home (e.g., [13,14]). Even if the number of newly infected individuals decreases, relaxing the interventions and resuming going out could cause a second wave of the spread of infections [15]. Because social lockdown is the only existing weapon to prevent the spread of the COVID-19 pandemic until vaccines are available to halt it, changing individuals’ behavior regarding staying at home is crucial [5].

In the early stages of a pandemic, effective communication of risk and preventive behaviors to the general public is one of the main public health strategies to reduce morbidity and mortality [16,17,18,19]. Protection motivation theory (PMT) [20,21] is one of the behavioral change theories that have been employed in such public health communications during pandemics [22,23]. PMT assumes that the motivation (i.e., intention) to protect oneself from danger is determined by two processes: threat appraisal and coping appraisal. Threat appraisal depends on the perceived severity of the health threat and on the perceived vulnerability to it. Coping appraisal depends on the perceived response efficacy (i.e., evaluation of whether the behavior is effective for alleviating the threat) and on perceived self-efficacy (i.e., evaluation of whether one will be able to carry out the behavior). PMT posits a positive linear relationship between the threat and coping appraisals and behavioral intention. In fact, previous studies showed that individuals’ perceived severity of the situation, vulnerability to the situation, response efficacy, and self-efficacy predicted their motivation to protect themselves during an influenza pandemic [22,23,24,25].

It is crucial to investigate factors that predict the behavior of staying at home during the COVID-19 pandemic, especially psychological factors that can be influenced through interventions. One previous study investigated the impact of online information regarding the intention to voluntarily self-isolate during the pandemic in Finland using PMT as a framework [26]. That study reported that perceived severity and self-efficacy had a positive impact on self-isolation intention [26]. However, no studies have investigated the relationship between PMT constructs and the intention to stay at home in Japan. Such an investigation is urgent under the situation where the Japanese state of emergency only included a few legally enforceable measures, as mentioned earlier. If psychological factors that predict the behavior of staying at home during the COVID-19 pandemic are revealed, the findings will contribute to more persuasive public health campaigns to encourage people to stay at home in Japan. Such public health campaigns are especially important in situations where vaccines are not yet available. We aimed to examine the psychological factors that predict the behavior of staying at home during the COVID-19 pandemic and social lockdown based on the PMT. Our research question was which construct of PMT predicted staying at home during the COVID-19 pandemic and social lockdown in Japan.

## 2. Materials and Methods

### 2.1. Study Design and Setting

This cross-sectional study was conducted from 9 May 2020 to 11 May 2020, when the state of emergency covered all prefectures in Japan. This cross-sectional study was conducted as part of another intervention study in Japan [27]. Participants in this cross-sectional study were the same as those in that intervention study. The sample size was determined for that intervention study [27]. Based on the effect size in a previous randomized controlled study [28], we estimated a small effect size (Cohen’s d = 0.20) in that intervention study [27]. We conducted a power analysis at an alpha error rate of 0.05 (two-tailed) and a beta error rate of 0.20. The power analysis indicated that 330 participants were required in each of the five intervention groups and one control group (i.e., 1980 participants in total). All participants in this study responded to all measures described below prior to the intervention in that study.

### 2.2. Participants and Procedures

The participants were recruited from people registered in a survey company database in Japan. E-mails were sent to 47,874 registered users. Of those, 5599 e-mail recipients responded to screening questions. Men and women aged 18–69 years were eligible to participate. The exclusion criteria were individuals who answered in the affirmative to screening questions asking if they are unable to cooperate with a study on the novel coronavirus infection being conducted by the University of Tokyo; if they could not go out due to illness or disability; if they had been diagnosed with a mental illness; or if they or their family members had been infected with COVID-19. In total, 1320 individuals were excluded from the screening questions.

A total of 4279 respondents who were determined to be eligible and consented to participate were invited to complete a web-based survey. The respondents answered questions on a computer or smartphone that was connected to the Internet. We stopped the invitations to complete the web-based survey once 3569 individuals had completed the survey. We set the number to 3569 individuals so as to recruit a larger sample of respondents than the required sample size, to avoid under-sampling owing to incomplete responses. A database of the survey company was used for allocation of sex, age, and residential area. Finally, 1980 participants were randomly selected from among the 3569 respondents. That random selection was conducted in keeping with the allocation according to the population composition ratio of Japan.

### 2.3. Ethical Considerations

The protocol was approved by the ethical review committee at the Graduate School of Medicine, University of Tokyo (number 2020032NI). All participants gave written informed consent in accordance with the Declaration of Helsinki.

### 2.4. Measures

#### 2.4.1. Sociodemographic Measures

The participants were asked for their sociodemographic information: gender, age, residential prefecture, educational background, and household income.

#### 2.4.2. Dependent Variable

Because there was no validated scale to assess behaviors of staying at home that was applicable to the present study, a measure was adapted from previous studies of influenza pandemic [29,30] and modified. Namely, from those measures of the previous studies [29,30], we excluded a question about the reduction of the amount of using public transport because those who live in city areas use public transport whereas those who live in rural areas use cars in Japan. We also excluded a question about taking time off work because it depended on workplaces whether to allow workers to commute to work under the Japanese state of emergency. Additionally, we changed a term of “the new flu” in the measures of those previous studies [29,30] to “the novel coronavirus” in the present study. In the present study, the participants responded to the following three questions on 1–10 scales ranging from “same as normal” to “not going out at all”: (1) Have you deliberately canceled or postponed plans such as “meeting people,” “eating out,” or “attending events” because of the novel coronavirus infection? (2) Have you deliberately reduced the time you spend shopping in stores outside your home because of the novel coronavirus infection? (3) Have you deliberately avoided crowded spaces because of the novel coronavirus infection? A mean score was calculated.

#### 2.4.3. Independent Variables 

For perceived severity, the participants responded to the following two questions: (1) How seriously do you think your health will be affected if you are infected with the novel coronavirus? (2) How serious do you think the social situation will be if the novel coronavirus spreads? For perceived vulnerability, participants responded to the following two questions: (1) How likely are you to be infected with the novel coronavirus? (2) How likely are you to be infected with the novel coronavirus when compared to someone of the same sex and age as you? These measures were adapted from previous studies [31,32]. We changed a term of “the new flu” in the measures of those previous studies [31,32] to “the novel coronavirus” in the present study. For perceived response efficacy, participants responded to the following explanation and three questions: Please share your thoughts on the effectiveness of staying at home to prevent the spread of novel coronavirus infection. (1) Do you think that you can save your life from the new coronavirus infection and prevent the spread of infection by canceling or postponing appointments such as “meeting people,” “eating out,” and “attending events”? (2) …. by reducing the time you spend shopping at stores outside your home? (3) …. by avoiding crowded spaces? For perceived self-efficacy, participants responded to the following explanation and three questions: Please share your thoughts regarding your confidence in the effectiveness of staying at home to prevent the spread of novel coronavirus infections. (1) Do you think that you can cancel or postpone appointments such as “meeting people,” “eating out,” and “attending events” because of the novel coronavirus infection? (2) Do you think you can reduce the time you spend shopping in stores outside your home ….? (3) Do you think you can avoid crowded spaces ….? Because there was no validated scale to assess response efficacy and self-efficacy regarding the COVID-19 pandemic, these measures were adapted from previous studies [29,30]. We changed a term of “the new flu” in the measures of those previous studies [29,30] to “the novel coronavirus” in the present study. The participants responded to these questions based on PMT on 1–6 scales on which the intervals indicated “extremely unlikely,” “unlikely,” “a little unlikely,” “a little likely,” “likely,” or “extremely likely” in ascending order. Mean scores were calculated for these measures. Higher scores indicated greater intention and perception.

### 2.5. Statistical Analysis

Cronbach’s α values were used to determine internal reliability of the measures. Descriptive statistics were used to describe participants’ sociodemographic information by summarizing categorical variables in percentage terms and giving mean ± SD for continuous variables. The residential areas of the participants were dichotomized to a binary variable indicating whether or not the area was within the “specified warning prefectures”. We examined the associations between sociodemographic information and staying at home using a two-sample t-test and a one-way ANOVA. We then calculated Pearson’s product-moment correlations to examine the simple association among study variables. We employed multiple regression analysis using perceived severity, vulnerability, response efficacy, self-efficacy, and sociodemographic measures as independent variables, and staying at home as a dependent variable. Additionally, we conducted subgroup analyses including only participants who lived in the “specified warning prefectures” using multiple regression analysis. In those multiple regression analyses, sociodemographic measures such as sex, age, educational background, and household income were included, based on published literature [16,23]. Participants’ residential area and whether they were living in one of the specified warning prefectures were also used as independent variables because they were presumed to be related to participants’ social behavior and social lockdown in Japan during the COVID-19 pandemic. A *p*-value of < 0.05 was set as significant in all statistical tests. All statistical analyses were performed using IBM SPSS Statistics for Windows, Version 21.0 (IBM Corp., Armonk, NY, USA).

## 3. Results

### 3.1. Descriptive Statistics

The mean values of the participants’ responses to the questions about staying at home were as follows: 7.25 (SD = 2.51) for canceling or postponing plans; 6.39 (SD = 2.51) for a reduction in time spent shopping; 7.67 (SD = 2.17) for the avoidance of crowded spaces. The Cronbach’s α values for the internal consistency of the responses to the questions were as follows: 0.835 for the behavior of staying at home; 0.480 for perceived severity; 0.875 for vulnerability; 0.921 for response efficacy; 0.853 for self-efficacy. Table 1 shows the participants’ characteristics and their associations with staying at home. As mentioned earlier, the participants’ sex, age, and place of residence were consistent with the population composition ratio in Japan. The behavior of staying at home was significantly associated with gender (*p* < 0.001), younger age (*p* = 0.002), residential area (*p* < 0.001), being in one of the specified warning prefectures (*p* < 0.001), higher educational background (*p* < 0.001), and larger household income (*p* < 0.001).

Table 2 shows the bivariate intercorrelations among study variables. Perceived severity, response efficacy, and self-efficacy showed positive correlations with staying at home and gender with weak to moderate associations (*p* < 0.001), respectively. However, perceived vulnerability did not. Perceived severity showed weak positive correlations with greater age, perceived vulnerability, response efficacy, and self-efficacy (*p* < 0.001, respectively). Perceived response efficacy showed a moderate positive correlation with self-efficacy (*p* < 0.001).

### 3.2. Regression Analysis

As shown in Table 3, when including all prefectures, perceived severity (Standardized β = 0.11, *p* < 0.001) and self-efficacy (Standardized β = 0.32, *p* < 0.001) significantly predicted more staying at home, controlling for gender, age, residential area, being in one of the specified warning prefectures, educational background, and household income. However, perceived vulnerability and response efficacy did not. The independent variables explained 21% of the variance in the dependent variable (adjusted R^2^ = 0.21).

The number of participants who lived in the “specified warning prefectures” was 1274. When including only participants who lived in the “specified warning prefectures”, perceived severity (Standardized β = 0.07, *p* = 0.006) and self-efficacy (Standardized β = 0.342, *p* < 0.001) significantly predicted more staying at home. However, perceived vulnerability and response efficacy did not.

## 4. Discussion

The regression analysis in the present study found that, of the four variables in the PMT, perceived severity and self-efficacy were significant predictors of staying at home during the COVID-19 pandemic and social lockdown in all Japanese prefectures, as well as the “specified warning prefectures”. This result was consistent with a previous study of voluntary self-isolation based on the PMT conducted during the COVID-19 pandemic in Finland, in which perceived severity and self-efficacy positively impacted self-isolation intention [26]. Perceived self-efficacy was a stronger predictor than perceived severity in the present study. This finding was consistent with previous studies of preventive behavior in the context of pandemic influenza, which showed that perceived self-efficacy is the strongest predictor of the intention of staying at home among the variables in the PMT [24,25]. These studies of pandemic influenza also showed that perceived severity was a significant predictor of staying at home [24,25], as found in this study.

However, perceived vulnerability and response efficacy were not significant predictors of staying at home in this study. This finding was inconsistent with previous studies [24,25]. As Table 2 shows, the perceived vulnerability of the participants in this study was not high: the mean value was 3.10, and participants’ response of 3 on a 1–6 scale was equivalent to a perception of being “a little unlikely” to be infected. This indicates that the participants in this study thought that they were unlikely to be infected and, therefore, that they were not motivated to restrain themselves from going out. Communication to increase perceived vulnerability is thus necessary. For example, health professionals and the mass media should place more emphasis on the fact that a characteristic of the novel coronavirus is that it is sometimes difficult to notice that someone is infected and, consequently, that it is hard to prevent infection if people come into contact with others. On the contrary, as Table 2 shows, the perceived response efficacy of the participants in this study was not low (mean = 4.44). This indicates that, even if the participants in this study thought that staying at home was somewhat effective in saving their lives and the spread of the disease, they were not motivated to restrain themselves from going out. This inference may indicate the difficulty of voluntary self-restraint with respect to going out.

The results of this study indicate that perceived severity rather than vulnerability should be increased to increase the threat appraisal, and that perceived self-efficacy rather than response efficacy should be improved to increase the coping appraisal, in order to encourage staying at home during the COVID-19 pandemic and social lockdown in Japan. To increase the perceived severity of infection with COVID-19, narrative communication can be a tool [33,34], e.g., a narrative message from a patient that conveys how severe the consequences of COVID-19 are [35]. Another example is a narrative message from a physician, stating that no treatment is available for COVID-19, that some patients rapidly develop severe symptoms, and that hospitals are overwhelmed [36]. Although social lockdown presumably evoked psychological reactance in many individuals [37], studies indicated that narrative messages obfuscate persuasive intent, subsequently reducing the psychological reactance and generating more persuasiveness than expository messages [38,39,40].

The heuristic rule of social norms—how others act in a given situation—is the self-protection system acquired by humans throughout evolutionary history [41] and has been shown to influence individuals’ judgment and behaviors [42]. In particular, it has been proposed that Japanese individuals has a collectivistic culture, and that there is a lot of pressure to conform to others in Japan [43]. Japanese individuals tend to be susceptible and influenced by information about how others act in a given situation [43]. Therefore, the heuristic rule of social norms can be used to increase perceived self-efficacy to stay at home [44]. For example, messages such as indicating the rate of reduction of movement of people, showing a picture of a downtown location without any people, and communicating narratives of people who spend time meaningfully at home, may increase self-efficacy to stay at home [44,45]. Additionally, providing behavioral alternatives, such as amusements at home or online social interaction, can also be a strategy to reduce psychological reactance and increase self-efficacy [46,47]. Public health experts, physicians and nurses, media workers, and individuals in general may be able to increase perceived severity and self-efficacy and subsequently encourage people to stay at home by disseminating such messages through the mass media and social networking services on the internet. Further, exposure to images of illness and death and news that inspires fear can increase anxiety and decrease confidence in coping with pandemic. Therefore, to avoid bias in news coverage, it will be crucial that public health professionals to work more effectively with the media; e.g., being readily accessible for journalists and providing reliable and useful information resources [48].

Finally, multiple regression analysis in this study found that female gender, younger age, and higher educational background were associated with more staying at home. The gender difference in this study was consistent with previous studies that females engaged in more precautionary behavior than their male counterparts during a swine flu pandemic [29,49] and pandemic COVID-19 [50], and with a literature review that indicated that females more often reported higher levels of risk as a concern than do males in general [51]. There has been discussion of social roles as a possible cause: due to their role as nurturer and care provider, females tend to avoid risks more than males, and due to their role as income earner, males tend to avoid risks less than females [51]. The reason why younger participants tended to stay at home may have been that they had more choices of home entertainment using the internet than older participants [52]. A higher educational background may contribute to the formation of enduring cognitive and emotional skills to foster health decisions, such as adopting behaviors to protect oneself against infectious diseases [53]. These results also should be considered for future research and practice to encourage people stay at home during pandemic.

Our study has several limitations. While the use of a panel database and a web-based survey had the advantage of allowing us to quickly recruit participants under the state of emergency, the selection bias of participants needs to be taken into account when interpreting the study results; participants in the present study did not represent the Japanese population. We assessed self-reported behavior rather than objectively measured behavior. The study results should be interpreted with caution because the measures used in the present study have not been validated and outcome scores may not appropriately reflect participants’ perception and behavior. The independent variables in this study did not include the response costs, as in some previous studies of infectious disease using PMT [54,55]. The cross-sectional design of this study constrains the ability to make causal inferences. Longitudinal research and randomized controlled studies will be necessary in the future to examine the temporal, causal relations. It is unclear to what extent the present findings are generalizable to populations other than the participants in this study.

## 5. Conclusions

During the COVID-19 pandemic and social lockdown in Japan, perceived severity and self-efficacy were the significant predictors of staying at home among the variables of the PMT. Perceived self-efficacy was a stronger predictor than perceived severity. Our findings indicate that, when encouraging people to stay at home during a pandemic, increasing perceived severity and self-efficacy by public health campaigns may consequently encourage people to stay at home. In future research, intervention studies will be needed to examine persuasive message content in terms of psychological factors, to encourage people to stay at home, e.g., determining whether intervention messages that increase perceived severity and self-efficacy encourage recipients to stay at home. We call for more studies to examine psychological factors that can encourage people to stay at home, especially in countries hit by second and third waves of infection. Public health experts, physicians and nurses, media workers, and influential individuals should disseminate messages that have been verified as influential by such studies. In that way, public health research, campaigns, and subsequent changes in individual behavior can help to slow the COVID-19 pandemic. Furthermore, examining psychological factors and messages to encourage people to stay at home based on the behavioral change theories will contribute to prevent the spread of other highly infectious diseases in the future as well.

## Figures and Tables

**Table 1:** Sociodemographic characteristics of the participants and their associations with staying at home (*n* = 1980).

Sociodemographic Characteristics	Overall (%)	Mean (SD) ^a^	*p*
Gender			
Men	49.7	6.76 (2.2)	<0.001 ^b^
Women	50.3	7.45 (1.9)	
Age			
18–29 years old	16.1	7.39 (1.9)	0.002 ^c^
Men	50.9		
30–39 years old	18.5	7.32 (2.0)	
Men	50.8		
40–49 years old	23.6	7.03 (2.2)	
Men	50.0		
50–59 years old	20.6	7.00 (2.1)	
Men	50.0		
60–69 years old	21.2	6.88 (2.2)	
Men	47.1		
Residential area			
Hokkaido	4.8	7.10 (2.2)	<0.001 ^c^
Tohoku	7.9	6.46 (2.3)	
Kanto	32.4	7.37 (1.9)	
Hokuriku and Chubu	17.9	6.89 (2.3)	
Kinki	16.7	7.22 (1.9)	
Chugoku and Shikoku	8.8	6.75 (2.3)	
Kyushu and Okinawa	11.5	7.22 (1.9)	
Specified warning prefectures			
Applicable	64.3	7.31 (1.9)	<0.001 ^b^
Not applicable	35.7	6.73 (2.3)	
Highest education			
Less than high school	1.7	6.40 (2.5)	<0.001 ^c^
High school graduate	26.0	6.79 (2.3)	
Some college	24.1	7.12 (2.2)	
College graduate	40.7	7.27 (1.9)	
Graduate school	7.5	7.40 (1.5)	
Household income ^d^			
Less than 2 million yen	9.2	6.57 (2.6)	<0.001 ^c^
2–6 million yen	43.9	7.07 (2.1)	
More than 6 million yen	37.2	7.34 (1.7)	
Unknown	9.6	6.87 (2.4)	

^a^ Standard deviation. ^b^ two-sample *t*-test. ^c^ a one-way ANOVA. ^d^ One US dollar is roughly equivalent to 100 yen.

**Table 2:** Correlations between variables (n = 1980).

.	Mean (SD)	1.	2.	3.	4.	5.	6.	7.	8.	9.
1. Staying at home	7.10 (2.08)									
2. Gender ^a^	-	0.17 **								
3. Age	-	−0.09 **	0.01							
4. Specified warning prefectures ^b^	-	0.13**	0.02	−0.01						
5. Education	-	0.11 **	−0.20 **	−0.18 **	0.13 **					
6. Income	-	0.06 *	−0.03	0.01	0.03	0.12 **				
7. Severity	4.33 (0.86)	0.18 **	0.07 **	0.12 **	−0.03	−0.07 **	−0.01			
8. Vulnerability	3.10 (0.91)	0.03	0.02	−0.08 **	0.03	0.02	0.02	0.26 **		
9. Response efficacy	4.44 (0.82)	0.29 **	0.13 **	−0.03	0.03	−0.01	0.03	0.23 **	−0.04 *	
10. Self-efficacy	4.68 (0.75)	0.39 **	0.15 **	−0.00	0.05*	−0.03	0.03	0.23 **	−0.03	0.64 **

* *p* < 0.05; ** *p* < 0.001. ^a^ The reference category is men. ^b^ The reference category is prefectures other than the specified warning prefectures.

**Table 3:** Regression analysis to predict staying at home (*n* = 1980) (Adjusted R^2^ = 0.21).

Variables	β	SE	95% CI	Std β	t	*p*
(Intercept)	−0.25	0.45	[−1.13, 0.63]		−0.55	0.580
Gender ^a^	0.54	0.09	[0.37, 0.71]	0.13	6.29	0.000
Age	−0.01	0.00	[−0.02, −0.01]	−0.09	−4.13	0.000
Residential area	0.04	0.03	[−0.01, 0.09]	0.03	1.49	0.137
Specified warning prefectures ^b^	0.47	0.09	[0.29, 0.64]	0.11	5.14	0.000
Education	0.25	0.05	[0.16, 0.33]	0.12	5.49	0.000
Income	0.10	0.05	[−0.01, 0.20]	0.04	1.81	0.070
Severity	0.27	0.05	[0.16, 0.37]	0.11	5.03	0.000
Vulnerability	−0.02	0.05	[−0.11, 0.08]	−0.01	−0.32	0.746
Response efficacy	0.10	0.07	[−0.03, 0.23]	0.04	1.46	0.145
Self-efficacy	0.88	0.07	[0.74, 1.02]	0.32	12.10	0.000

SE = Standard Error. CI = Confidence Interval [lower-bound, upper-bound]. Std β = Standardized β. ^a^ The reference category is men. ^b^ The reference category is prefectures other than the specified warning prefectures.
